# Structural analysis of the complex between influenza B nucleoprotein and human importin-α

**DOI:** 10.1038/s41598-017-17458-z

**Published:** 2017-12-07

**Authors:** Alice Labaronne, Sigrid Milles, Amélie Donchet, Malene Ringkjøbing Jensen, Martin Blackledge, Jean-Marie Bourhis, Rob W. H. Ruigrok, Thibaut Crépin

**Affiliations:** University Grenoble Alpes, CNRS, CEA, IBS, F-38000 Grenoble, France

## Abstract

Influenza viruses are negative strand RNA viruses that replicate in the nucleus of the cell. The viral nucleoprotein (NP) is the major component of the viral ribonucleoprotein. In this paper we show that the NP of influenza B has a long N-terminal tail of 70 residues with intrinsic flexibility. This tail contains the Nuclear Location Signal (NLS). The nuclear trafficking of the viral components mobilizes cellular import factors at different stages, making these host-pathogen interactions promising targets for new therapeutics. NP is imported into the nucleus by the importin-α/β pathway, through a direct interaction with importin-α isoforms. Here we provide a combined nuclear magnetic resonance and small-angle X-ray scattering (NMR/SAXS) analysis to describe the dynamics of the interaction between influenza B NP and the human importin-α. The NP of influenza B does not have a single NLS nor a bipartite NLS but our results suggest that the tail harbors several adjacent NLS sequences, located between residues 30 and 71.

## Introduction

Both transcription and replication of influenza virus take place in the nucleus of the infected cell. To ensure these two mechanisms, a number of viral proteins have to be transported from the cytoplasm to the nucleus^1^. These are in particular the three subunits of the RNA-dependent RNA-polymerase of the virus and the nucleoprotein (NP), which is the major protein of the ribonucleoproteins (RNPs) that binds the viral RNA^2^ and has been shown to be essential for viral proliferation.

Transport of large proteins (>40 kDa) into the nucleus is generally mediated by the presence of a nuclear localization signal (NLS). NLSs are often made by short motifs with basic amino acids^3–6^, which mediate the interaction with proteins of the karyopherin family, in particular different importin-α variants^7,8^. By specifically recognizing NLSs of cargo proteins, importin-alphas act as an adaptor protein for the nuclear transport, through a complex with an importin-β receptor^9^. Once inside the nucleus, the heterotrimeric importin-β:importin-α:cargo complex interacts with Ran:GTP, resulting in dissociation of the cargo from its carrier concomitant to the hydrolysis of GTP^8,10,11^.

Two putative NLSs have been described in influenza A NP (A/NP): NLS1 is a non-classical (ncNLS) motif in the first 14 amino acids of the unfolded N-terminal region, while NLS2 consists of residues 198–216 located at the surface in the middle of the protein^12–18^. Recently, two crystal structures of the NLSs of influenza virus NP bound to importin-α have been published: the structure of NLS1 (residues 3–14) bound to the minor NLS-binding pocket of importin-α and the structure of NLS2 (residues 213–216) interacting with the major pocket of importin-α^19,20^. Both NLSs bind to importin-α with high dissociation constants (K_d_s between 2 and 5 µM for NLS1 and 70 µM for NLS2). Wu and coworkers suggest that importin-α can bind to both sites, either on the same monomer of NP or on two different protomers inside the NP-trimer, so that the synergy of the two sites is strong enough for the transport of NP into the nucleus^20^. Phosphorylation sites have been identified in the NLS1 of A/NP (S/T3, S9 and Y10) suggesting that phosphorylation at these sites prevent the interaction of NLS1 with importin-α^21,22^.

**Figure 1:**
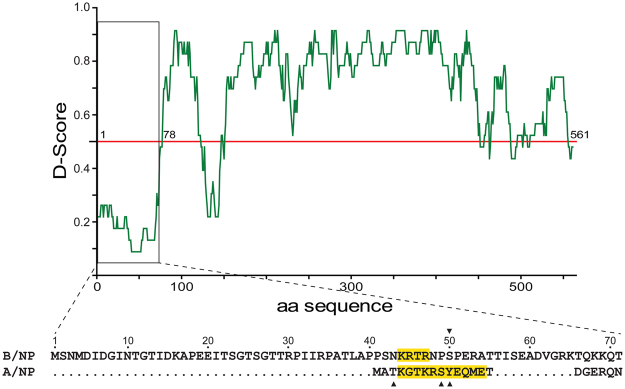
Computational analysis of B/NP. The sequences of the N-termini of B/NP (strain B/Memphis/13/03) and A/NP (strain A/WSN/1933) have been aligned using Clustal W^71^. On the sequence alignment, the putative NLSs motifs of the two proteins are highlighted in yellow. The black triangles show the phosphorylation sites of each protein^72^. D-score is an algorithm to find structured and disordered regions in proteins^25^. The prediction is based on 22 predictor web servers. The value 1.0 means that the protein is fully ordered and 0.0 means the peptide is fully disordered.

Except in their respective oligomeric state, the overall structures of A/NP and B/NP are highly similar^15,23,24^. The main difference between the two proteins lies in their N-terminal extremities: A/NP has an N-terminal tail of about 20 residues with NLS1 whereas B/NP has a 70-residue N-terminal tail (Fig. 1). Both were present in the protein used for the crystallogenesis but were not observed in the X-ray structures. We used several algorithms^25^ to predict the probability of B/NP_TAIL_ to contain secondary structures. Figure 1 gives the D-score (disordered score) of B/NP showing that the first 70 residues are most likely disordered without any stable secondary structures. However, the location of the NLS in B/NP cannot be clearly identified. Stevens and Barclay have shown that deletions of the N-terminus up to residue 69 do not impair the nuclear accumulation of B/NP^26^ whereas others suggested that residues 44-KRTR-47 is the putative NLS^27–29^.

With this paper we present biochemical and biophysical data that demonstrate that the 70 N-terminal amino acids of B/NP are intrinsically disordered. We show by size exclusion chromatography, NMR and SAXS, that this tail binds to human importin-α7, with a *K*_*d*_ value for this interaction estimated by isothermal titration calorimetry. The precise interaction site of the NP tail (NP_TAIL_) in complex with importin-α was mapped using NMR spectroscopy, revealing the extent of the interacting region.

## Results

### The N-terminus of the B/NP is disordered

**Figure 2:**
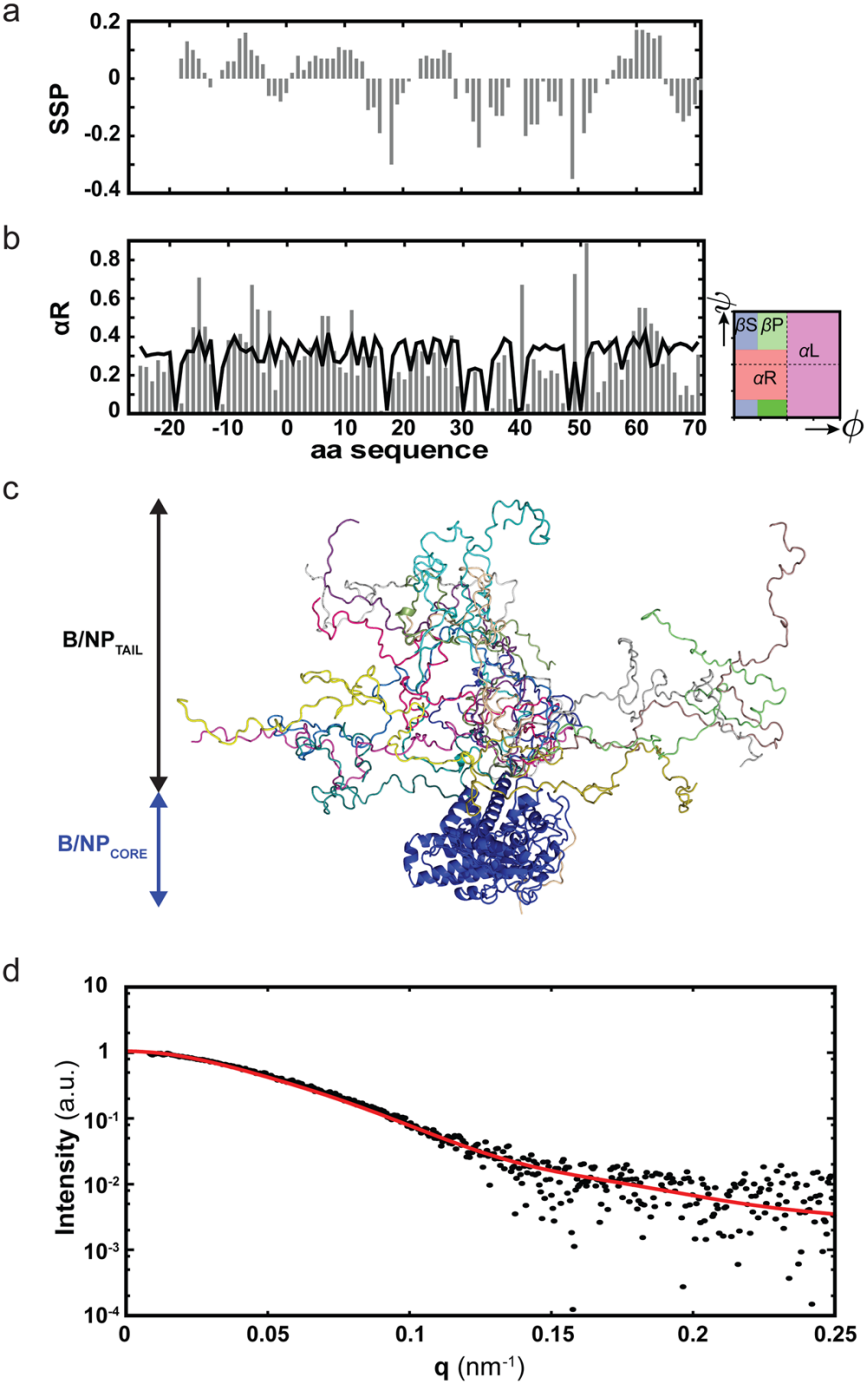
Conformational propensities of B/NP_TAIL_. (**a**) Secondary structure propensities (SSPs) calculated from Cα chemical shifts as obtained from the assignment of the N-terminally tagged protein. Residues are numbered with 1 starting at the methionine of B/NP_TAIL_. SSP scales from −1 to 1. SSP > 0: α-helical propensity; SSP < 0: propensity for extended structure. (**b**) Ramachandran plot showing regions of β-sheet (βS), poly-proline (βP), right (αR) and left handed (αL) helical conformations (right) and corresponding αR propensities (grey bars) as derived from a set of 200 conformers describing the structural propensities of B/NP_TAIL_ in a combination of Flexible-Meccano^30^ and ASTEROIDS^31^ using chemical shifts. Supplementary Figure 1 details the data obtained for αL, αR, βP and βS. The black trace reflects the propensities of a statistical coil ensemble without selection based on experimental data. (**c**) Conformational ensemble (10 of 1000 conformers are shown) of full length B/NP calculated from the crystal structure of B/NP_CORE_ (PDB 3TJ0^23^) and Flexible-Meccano using the backbone dihedral angles as obtained for B/NP_TAIL_ to describe the N-terminal intrinsically disordered region. The 10 different conformations of B/NP_TAIL_ are shown in different colours with the core of B/NP in blue. (**d**) Averaged SAXS curves calculated from the ensemble as described in (**c**) (red line) overlaid with the experimental SAXS curve of B/NP full length (black dots).

We aimed to characterise the structural propensities of the first 70 N-terminal residues (B/NP_TAIL_) using NMR spectroscopy and we therefore obtained the complete backbone resonance assignment of B/NP_TAIL_. We calculated secondary structure propensities (SSPs) based on experimental Cα and Cβ chemical shifts, which indicate that no strong propensities for either α-helical or extended structure exist (Fig. 2a). We then developed a multi-conformational model of B/NP_TAIL_ using Flexible-Meccano^30^ and the genetic algorithm ASTEROIDS^31^ that allowed us to select sub-ensembles of 200 conformers on the basis of the experimental ^13^C, ^15^N and ^1^H chemical shifts (Fig. 2b and Supplementary Figure 1). The obtained conformational ensembles describe B/NP_TAIL_ as a protein behaving much like a statistical coil, with the exception of a region starting around residue 59 that had a slightly increased propensity to form right handed α-helices as compared to random coil. The backbone dihedral angles describing B/NP_TAIL_ were then used to calculate a model of full length B/NP using the crystal structure of the folded domain^23^ and Flexible-Meccano^30^ to add the intrinsically disordered tail (Fig. 2c). The experimental SAXS curve of full length B/NP was in reasonable agreement (chi^2^ below 1) with this conformational ensemble (Fig. 2d).

### Binding of B/NP and importin-α7 analysed by size-exclusion chromatography

**Figure 3:**
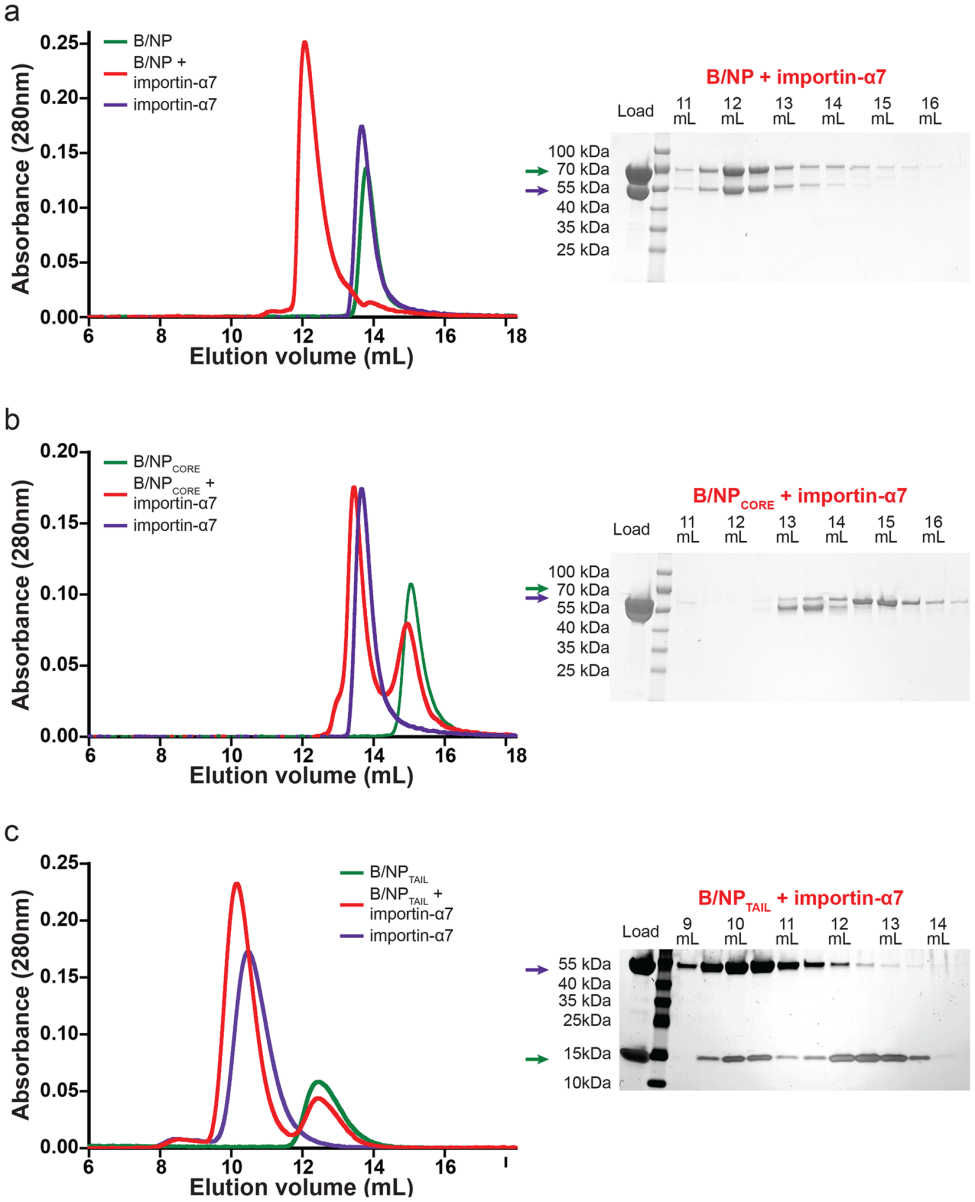
Interaction of B/NP with importin-α7. The binding experiments with importin-α7 were performed by size elution chromatography, respectively using a Superdex^TM^ 200 increase 10/300GL column for (**a**) B/NP and (**b**) B/NP_CORE_ and a Superdex^TM^ 75 10/300GL column for (**c**) B/NP_TAIL_. On the left are the elution profiles of the binding tests and on the right are the corresponding coomassie blue colored SDS-PAGE gels (Tris-Glycine, 4–20% polyacrylamide) for the mixture between the two partners. The ratio used for these experiments are 1.2:1 for B/NP:importin-α7 and B/NP_CORE_:importin-α7 and 2:1 B/NP_TAIL_:importin-α7. The SDS-PAGE gels for the controls are shown in Supplementary Figure 2.

We have previously shown that, under the same experimental conditions as used here (*i.e*. 20 mM Tris-HCl pH 7.5; 150 mM NaCl), B/NP is mainly monomeric^32^. B/NP (residues 1 to 561) and B/NP_CORE_ (residues 71 to 561) were injected separately or in complex with importin-α7, on a size exclusion chromatography column (Superdex^TM^ increase 200 10/300 GL column). B/NP and importin-α7 alone are both eluted as a single peak, with a respective elution volume of 13.8 mL and 13.7 mL (Fig. 3a and Supplementary Figure 2). When the two proteins are mixed before injection, the elution profile presents a single peak at 12 mL and the SDS-PAGE confirms the presence of the two proteins in the peak. For B/NP_CORE_ and importin-α7 (Fig. 3b and Supplementary Figure 2), both samples are eluted as a single peak, respectively at 15 mL and 13.7 mL. By a SEC-MALLS-RI experiment, we confirmed the molecular mass of B/NP_CORE_ (Supplementary Figure 3). When B/NP_CORE_ and importin-α7 are mixed before injection, we observed an elution of the two proteins in two separate peaks and no shift in the elution volume is observed. We can conclude that without the N-terminal tail on NP, the two proteins do not interact under the experimental conditions. For B/NP_TAIL_ and importin-α7 (Fig. 3c and Supplementary Figure 2), the samples were injected on a Superdex^TM^ 75 10/300 GL column with an excess of B/NP_TAIL_. The peak eluted at 12.5 mL corresponds to the B/NP_TAIL_ and the SDS-PAGE gel confirms the presence of the protein contained in that elution peak (band at 15 kDa). The elution peak at 10.5 mL corresponds to importin-α7 alone and the peak at 10.1 mL corresponds to the complex between B/NP_TAIL_ and importin-α7. These results confirm that importin-α7 binds the full-length influenza B nucleoprotein and its N-terminal tail but not B/NP_CORE_.

### Analysis of the importin-α binding site on B/NP_TAIL_ by NMR

**Figure 4:**
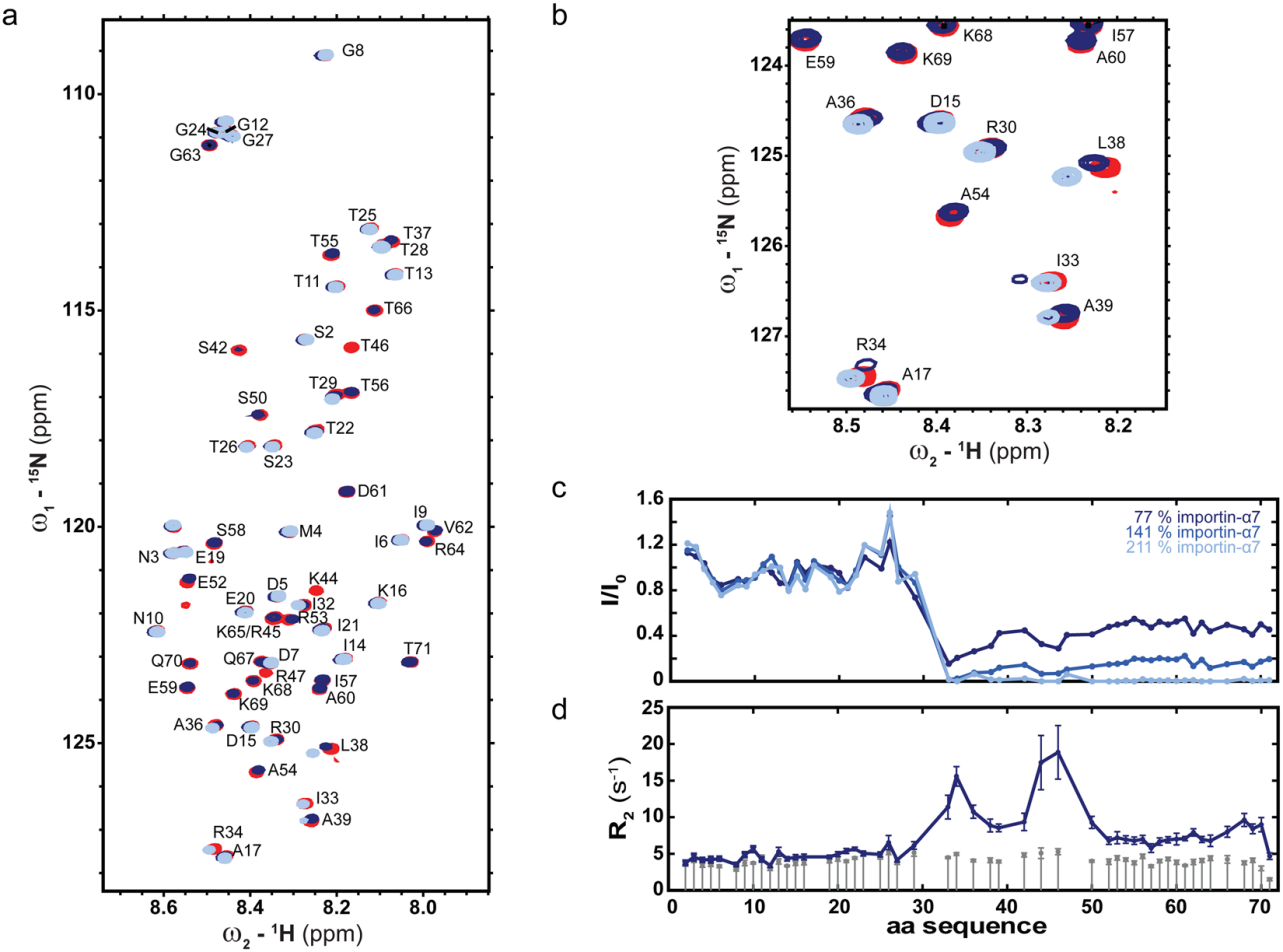
Mapping of B/NP_TAIL_:importin-α7 interaction by NMR. (**a**) ^1^H-^15^N HSQC spectra of B/NP_TAIL_ alone (red) and in the presence of 77% (dark blue) and 211% importin-α7 (light blue) respectively. The assignment is displayed as one letter code. (**b**) Zoom into the ^1^H-^15^N HSQC spectra from (**a**), demonstrating localized chemical shift changes. (**c**) Intensity ratio of bound as compared to unbound B/NP_TAIL_. B/NP_TAIL_:importin-α7 ratios were 1:0.77, 1:1.41, and 1:2.11 (dark to light blue). Intensities were normalized to 1 at the maximum intensity ratio. (**d**) ^15^N transverse relaxation rates (R_2_) were measured in the absence (grey bars) and presence (dark blue line) of 77% importin-α at a ^1^H frequency of 950 MHz. All measurements were done at 25 °C.

In order to precisely map the residues of B/NP_TAIL_ that are involved in the interaction with importin-α, we titrated ^15^N labelled B/NP_TAIL_ with unlabelled importin-α7 and measured ^1^H-^15^N HSQC spectra at each titration step. Peak intensities were extracted and compared to those of the unbound B/NP_TAIL_ (Fig. 4a and b). Interestingly, the interaction between B/NP_TAIL_ and importin-α involves all residues of the disordered tail between amino acids 30 and 71 (Fig. 4c). This may be relevant to the recent observation that the cytoplasmic fraction of B/NP is directly proportional to truncations made on its N-terminal tail^28^.

We then analyzed B/NP_TAIL_ for the presence of other putative NLS motifs that could explain this interaction using the eukaryotic linear motif database (ELM)^33^. A classical NLS is predicted, starting from residue 41. The analysis of the sequence by a prediction server that is specialized for the prediction of composite motifs, cNLS Mapper^34^ predicts indeed a bipartite NLS between residues 41 and 68. Interestingly the region of residues 41 to 68 also contains the region with increased helical propensity – a frequent observation in case small recognition motifs are encoded in an otherwise disordered chain. Residues 30–39, however, are additionally involved in the interaction and show characteristics of two conformational states in slow exchange on the NMR chemical shift time scale, visualized by peaks splitting in the course of the titration. While the ncNLS of influenza A has been shown to bind to the minor groove of importin-α1^19,20^, bipartite NLS motifs are thought to bind both the major and the minor binding site^35^. The extended NLS region in B/NP may explain the convolved dynamic features observed in the interaction, switching between slow exchange from residues 30–39 and fast or intermediate exchange in the region of residues 41–50 (Fig. 4d).

### SAXS analysis

**Figure 5:**
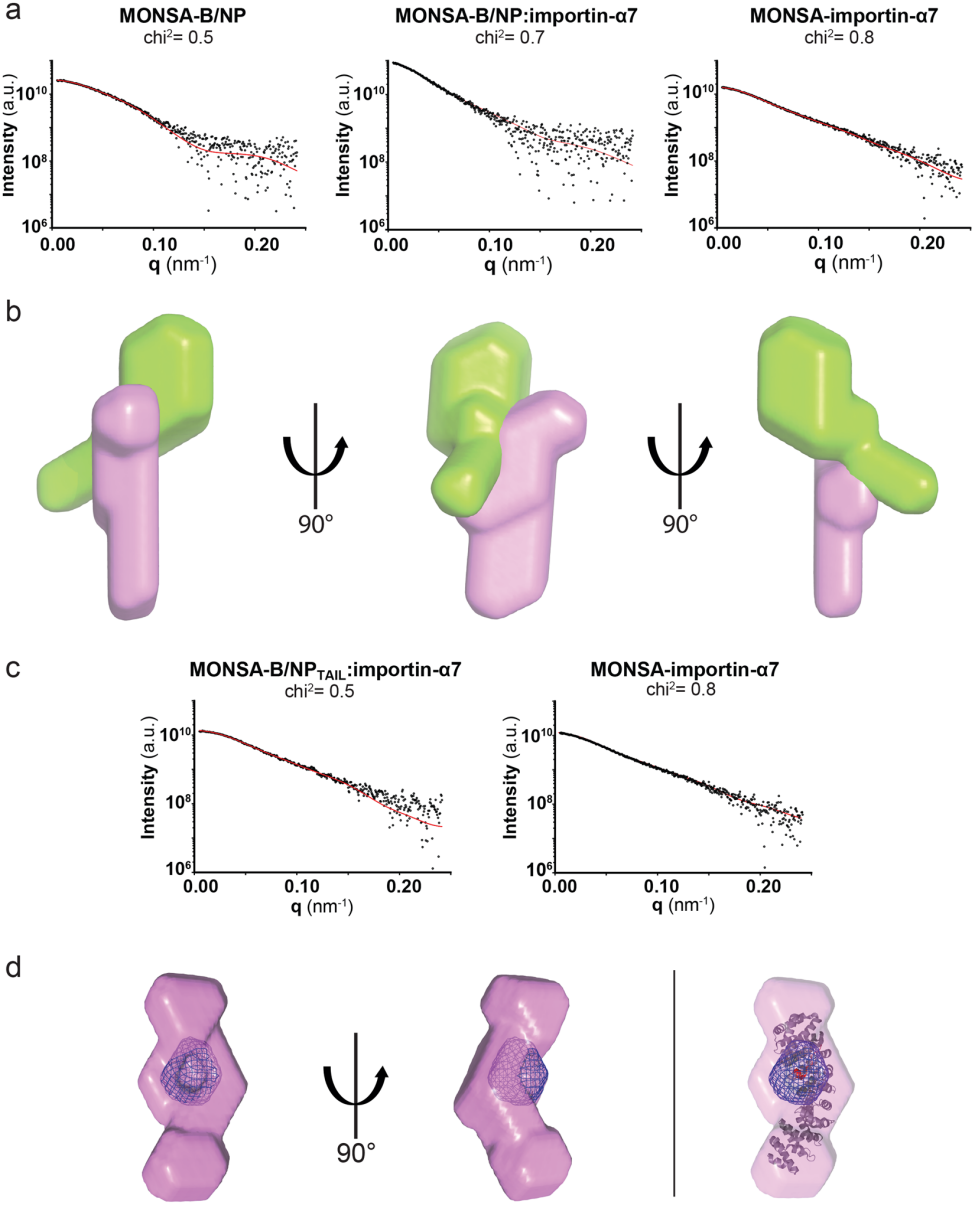
Modelling of the B/NP:importin-α7 interaction by SAXS. (**a**) Observed scattering (dots) profiles for B/NP alone, B/NP:Imp-α7 and human Imp-α7 alone (from left to right) with MONSA-generated fits (solid red lines). (**b**) Low-resolution structure of the B/NP:Imp-α7 complex with B/NP in green and importin-α7 in pink. (**c**) Observed scattering (dots) profiles for B/NP_TAIL_:Imp-α7 and Imp-α7 alone (from left to right) with MONSA-generated fits (solid red lines). (**d**) 90°-rotation view of the B/NP_TAIL_:Imp-α7 low-resolution structure with B/NP_TAIL_ shown as a blue grid density and human importin-α7 in pink. The right part corresponds to the docking of the X-Ray structure of A/NP_TAIL_:Imp-α1(mouse) complex (PDB id: 4ZDU;^19^) in the MONSA envelop obtained for B/NP_TAIL_:Imp-α7.

We used in-line SEC-SAXS analysis for the characterization in solution of B/NP, importin-α7 and the complexes B/NP:importin-α7 and B/NP_TAIL_:importin-α7. Each dataset has been processed individually and provides coherent statistics (Table 1). By using the volume of correlation determination methods^36^, the molecular weights (M_R_) were estimated (Table 1) in accordance with the respective calculated masses from the sequences. The hydrodynamic radius (R_g_) and D_max_ values of each sample are listed in Table 1 and the Guinier plots are shown in Supplementary Figure 4. The Guinier plot estimates two essential SAXS parameters, the size (through the R_g_) and the mass (through the extrapolated intensity at zero scattering angle (I(0))) of the analyzed sample. An absence of linearity in the Guinier plot reflects the presence of aggregates and/or attractive/repulsive interactions between the scattering particles. We have combined the processing of the dataset in order to further localize the B/NP NLS binding site on the importin-α7. The program MONSA builds *ab initio* shapes using the scattering curves for the complexes together with those of their individual components^37,38^. While the shapes obtained from a protein containing an intrinsically disordered tail will not be expected to provide realistic shapes (compare Fig. 2c with Fig. 5), we still expect this analysis to provide qualitative information on the orientation of the two proteins when bound to each other. For the B/NP:importin-α7 complex (Fig. 5a and b), the resulting shapes seem to be made by two parts: a globular green domain with an extension and a more flat entity (purple). We can postulate that the extension of the globular green domain corresponds to B/NP_TAIL_ which we have shown to be flexible in solution, and the globular part is then B/NP_CORE_. In this hypothesis, the flat entity would be the importin-α7. This is a reasonable description of the *ab initio* shapes as the NLS sequence is located within NP_TAIL_ that would be, in that case, the main contact between the two partners of the complex. For the shape corresponding to the importin-α7, the contact seems to occur with only one third of the shape, meaning that only a part of the protein is involved in the interaction.

**Table 1:** SAXS data collection and scattering-derived parameters.

	B/NP	importin-α7	B/NP:importin-α7	B/NP_TAIL_:importin-α7
**Data collection parameters**				
Instrument	ESRF - BM29			
Beam size at sample (µm)	700 × 700			
Wavelength (Å)	0.9919			
q range (Å^−1^)	0.25–50			
Detector	Pilatus 1 M			
Detector distance	2.867			
Exposure (s per image)	1			
Column	Superdex^TM^ increase 200 5/150 GL			
Flow rate (mL.min^−1^)	0.5			
Injected sample concentration (mg.mL^−1^)	10			
Injected volume (µL)	50			
Temperature (K)	293			
**Structural parameters**				
R_g_ (Å) [from P(r)]	33.5	38.3	47.5	35.8
R_g_ (Å) [from Guinier]*	34.0	37.8	47.2	34.4
D_max_ (Å)	119	133.5	163.6	120.3
Porod volume estimate (Å^3^)	109 290	81 420	246 570	83 120
Molecular mass M_R_ (kDa) from Rambo	63	54	151	57
Calculated M_R_ (kDa) from sequence	61.6	57.0	118.6	64.5
**Software employed**				
Primary Data reduction	Primus			
Data processing	Primus			
*Ab initio* analysis	MONSA			
3D graphics representation	PyMOL			

*The Guinier plots are shown on Supplementary Figure 4.

In order to get a closer view for the localization of the NLS major binding site on the importin-α7, we have generated *ab initio* shapes with MONSA, corresponding to B/NP_TAIL_ bound to the importin-α7 (Fig. 5c and d). An extra density corresponding to B/NP_TAIL_ can be observed in one third of the shape of the importin-α7 (blue ball on Fig. 5d). This observation is coherent with the shapes obtained for the B/NP:importin-α7 complex. The X-ray model of the complex between the mouse importin-α1 and the NLS1 of A/NP (PDB id: 4ZDU) has been docked in our *ab initio* SAXS envelope (Fig. 5d on the right, red part). The superimposition shows that the NLS of A/NP fits in the additional density of our shape.

### Determination of the affinity constant between B/NP_TAIL_ and importin-α with ITC

**Figure 6:**
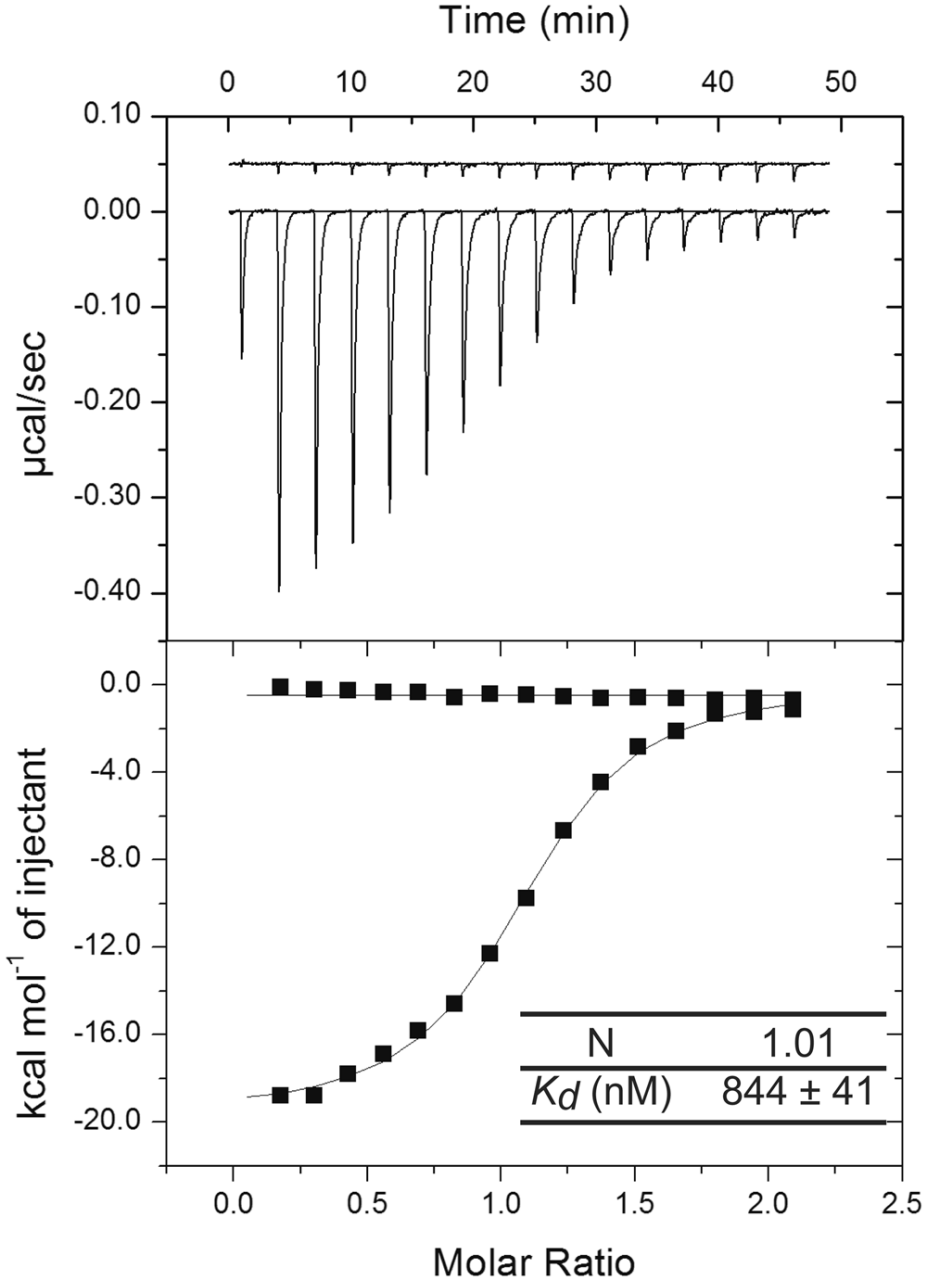
ITC determination of the binding thermodynamics of B/NP_TAIL_ to importin-α7. Titration of 150 µM of importin-α7 into a solution of 15 mM of B/NP_TAIL_. The experiments were performed in 20 mM Tris-HCl pH 7.5; 150 mM NaCl at 25 °C in triplicate.

We have determined the binding affinity of B/NP_TAIL_ to importin-α7 using ITC (isothermal calorimetry). The experiment was done in triplicates. One of the titration curves is shown in Fig. 6 and all the data are compiled in a table on the same figure. B/NP_TAIL_ binds human importin-α7 with a *K*_*d*_ of 844 nM, similar to the affinity measured between A/NP and the mouse importin-α1 (*K*_*d*_ between 2 and 5 µM)^19,20^. We tried to repeat the experiment with the full-length B/NP, but because of its tendency to adopt tetramers under these conditions, a meaningful interpretation of the ITC data could not be obtained.

## Discussion

This paper shows that the long N-terminal region of influenza B nucleoprotein is disordered. Figure 2c shows that the B/NP_TAIL_ samples a large space. The long N-terminal tail of influenza B NP can therefore change how the B/RNPs (RNPs from influenza B virus) pack together in comparison to what has been shown for influenza A^39^. CryoEM of paramyxoviruses like Sendai or measles viruses^40,41^, which have very long N_TAILS_^42,43^ shows that the nucleocapsids take a large volume inside the viral particle, unlike the nucleocapsids from rhabdovirus particles, because the nucleocapsids of rabies and VSV have no N_TAILS_^44,45^. In the influenza A viral particle the RNPs form the oval shape of the virus with a tight enwrapping of the envelope^46,47^ whereas the particles from Influenza virus B seem to be more loose and spherical^48–50^.

Like the shorter N-terminal end of influenza A, the B/NP_TAIL_ carries the NLS of the protein. Whereas the non-classical NLS1 of A/NP is rather short with about 11 residues, the NLS of B/NP show three overlapping NLS sequences from residue 30 up to the end of the tail at residue 71. This result could explain why stepwise shortening of the tail gradually decreases the propensity of NP to go into the nucleus implicating a longer stretch in the interaction with importin-α rather than just a 4 residue linear motif between 44 and 47^28^. The complex between importin-α7 and B/NP was studied with SAXS. The analysis shows the volumes of the two proteins. Although the SAXS envelope of a disordered chain cannot give a single structure for B/N_TAIL_ the complex shows that importin-α binds two thirds of the tail, concordant with the results from NMR, and no other parts of B/NP.

For the complex between the B/N_TAIL_ and importin-α we used importin-α7. For efficient virus replication *in vivo*, avian influenza viruses depend on importin-α3, whereas mammalian viruses depend on importin-α7^51^. However, all importin-alphas are very similar in their sequence and their structures and a specific importin-α can bind a very large range of proteins. The affinity between B/N_TAIL_ and importin-α is rather low (*K*_*d*_ around 900 nM) as measured with ITC and close to the affinity between the A/N_TAIL_ and mouse importin-α1 (1.7 µM). This affinity is much lower than the affinity between the influenza polymerase protein PB2 and importin-α which has a *K*_*d*_ value of around 5 nM^52^. Maybe one of the reasons is that the infected cell makes a lot of NP and only very little PB2, at least in earlier hours of the infection^53,54^.

Despite its intrinsic flexibility, the disordered N-terminal tail of NP may present a viable target for inhibition of viral infection, due to its essential role in nuclear import. Although the development of inhibitory molecules for intrinsically disordered targets represents a significant challenge^55,56^, recent advances have demonstrated that these flexible domains can be targeted^57–60^ and biophysical descriptions of the behavior of linear motifs such as the NLS, and their complex interaction modes, will no doubt aid in the conception of rational peptide or small-molecule based strategies.

## Methods

### Expression and Purification

The cloning of the human importin-α7 (KPNA6; Uniprot O60684) DNA coding sequence was described in^52^. The full length B/NP (B/NP_1-560_ called B/NP), its N-terminus (B/NP_1-70_ called B/NP_TAIL_) and its core (B/NP_71-560_ called B/NP_CORE_), were cloned in the pETM11 vector (EMBL) to express N-terminal His-tag TEV protease-cleavable constructs. *Escherichia coli* BL21 (DE3) cells transformed with the corresponding plasmids were induced 12 hours by adding 0.3 mM isopropyl-β-D-thiogalactopyranoside (IPTG) at 18 °C and collected by centrifugation. For B/NP constructs, pellets were resuspended and sonicated in lysis buffer composed of 50 mM Tris-HCl pH 7.5, 300 mM NaCl, 1 M NDSB201 (Sigma), 5 mM β-mercaptoethanol (β-ME) and cOmplete EDTA-free protease inhibitor cocktail (Roche). For importin-α7, pellets were resuspended and sonicated in lysis buffer composed of 50 mM Tris-HCl pH 8, 500 mM NaCl, 1 mM β-ME and cOmplete EDTA-free protease inhibitor cocktail. Purifications were performed at room temperature. Proteins were purified by Ni^2+^ affinity chromatography (Ni-NTA, Qiagen). For the B/NP and the B/NP_CORE_, this step was followed by a heparin column (GE-Healthcare). Heparin elution fractions or nickel elution fractions were dialyzed overnight at 4 °C with TEV (1/100) against 20 mM Tris-HCl pH 7.5 at 150 mM, 5 mM β-ME and 20 mM imidazol, followed by Ni^2+^ affinity chromatography to remove the His-tag and the TEV protease. The proteins were then purified by size-exclusion chromatography using a Superdex^TM^ 200 increase 10/300 GL column (GE healthcare). For ITC and NMR, the His-tag of B/NP_TAIL_ was removed and purified by size-exclusion chromatography (Superdex^TM^ 75 10/300 GL column; GE Healthcare). The purities of the samples were confirmed by SDS-PAGE. Proteins were concentrated by centrifugation (Amicon concentrators with cutoff of 3 and/or 10 kDa). Protein concentrations were determined using the extinction molar coefficient at 280 nm ε = 46 785 M^−1^.cm^−1^ for importin-α7, ε = 24 995 M^−1^.cm^−1^ for B/NP and B/NP_CORE_ and ε = 2980 M^−1^.cm^−1^ for B/NP_TAIL_. For B/NP_TAIL_ without the his-tag, the concentration was determined using a bicinchoninic acid protein assay (BCA protein assay kit, Pierce).

### NMR experiments

Samples for NMR spectroscopy were produced in M9 minimal medium containing MEM vitamins (Gibco). For producing ^13^C, ^15^N proteins the medium was supplemented with 1.0 g.L^−1^ of ^15^NH_4_Cl (Cambridge Isotope Laboratories, INC; USA) and 2.0 g.L^−1^ of D-glucose U-^13^C6 (euriso-top; France). Purification protocol was the same as above and all NMR experiments were performed in 20 mM Bis-Tris buffer pH 6.5, 50 mM NaCl.

Spectral assignment of ^13^C, ^15^N B/NP_TAIL_ was obtained from a set of BEST-type triple resonance spectra: HNCO, intra-residue HN(CA)CO, HN(CO)CA, intra-residue HNCA, HN(COCA)CB, and intra-residue HN(CA)CB^61^. All assignment spectra were recorded at a ^1^H frequency of 700 MHz and at 5 °C. The spectra were processed with NMRPipe^62^ and automatic assignment was done with the program MARS^63^ and manually verified. Secondary chemical shifts were calculated using the random coil values from refDB^64^.

A multi-conformational model of B/NP_TAIL_ was calculated based on the chemical shifts as obtained from the assignment using a combination of Flexible-Meccano and the genetic algorithm ASTEROIDS. Five times 200 conformers were selected from a statistical coil ensemble of 10,000 conformers^31^. A new ensemble of 8,500 conformers was generated based on the φ and ψ angles of the selected conformers, and supplemented with 1500 structures from the initial ensemble. This ensemble was subjected to another round of ASTEROIDS selection and this iteration was repeated four times^30^ until convergence was achieved with respect to the experimental chemical shifts.

For titration of ^15^N labelled B/NP_TAIL_ with importin-α, ^1^H-^15^N HSQC spectra of 71 μM B/NP_TAIL_ were recorded at increasing concentrations of importin-α (55, 100, 150 μM) at 25 °C and a ^1^H frequency of 850 MHz. ^15^N R_2_ (CPMG) were recorded at a ^1^H frequency of 950 MHz in the absence and presence of 55 μM importin-α at 25 °C using delay times of 4, 8, 20, 40, and 68 ms to measure the magnetization decay^65^. The time point at 8 ms was repeated for error estimation. Assignment experiments were performed with the purification tag present, whereas all interaction experiments with importin-α were performed with the purification tag cleaved off B/NP_TAIL_.

### Interaction assays by size exclusion chromatography

All size exclusion chromatography (SEC) experiments were performed in 20 mM Tris-HCl pH 7.5, 150 mM NaCl and 5 mM β-ME using a Superdex^TM^ 200 increase 10/300GL column for B/NP and B/NP_CORE_ and a Superdex^TM^ 75 10/300GL column for B/NP_TAIL_. 300 μL of each sample (30 μM B/NP; 30 μM B/NP_CORE_; 60 μM B/NP_TAIL_; 25 μM importin-α7; 30 μM B/NP + 25 μM importin-α7; 30 μM B/NP_CORE_ + 25 μM importin-α7; 50 μM B/NP_TAIL_ + 25 μM importin-α7) were incubated 1 hour at room temperature before injection.

### SAXS analysis

All datasets were collected on BM29 (ESRF). For in-line SEC-SAXS, the experimental setup consists of High Pressure Liquid Chromatography (HPLC) system connected to an analytical Superdex^TM^ increase 200 5/150 GL column (GE Healthcare) followed down-stream by SAXS sample capillary. SAXS measurements were performed every second with a Pilatus 1 M detector (distance of 2.87 m) allowing a q range of 0.03 to 4.5 nm with a wavelength of 0.01 nm.

Experimental curves were subtracted and analyzed using Primus (ATSAS programs suite)^66^. To verify the molecular mass, the Rambo and Tainer method was used^36,67^. R_g_ predictions using Guinier extrapolation were plotted against the elution volume to select the most monodisperse part of the protein elution peak. SAXS datasets within this zone were scaled and averaged to produce one unique I(q) curve. Distance distribution functions p(r) were calculated using the program GNOM^68^. The ab-initio models were generated by MONSA using when available, the individual data sets in order to fit them simultaneously^37^. Homologue PDB structure comparison was assessed using Crysol^69^. Homologue structure fitting within the DAMAVER envelope was performed with PyMOL^70^ and curve representations using Graphpad (Prism). SAXS curves with B/NP alone contained the poly-His tag, which is cleaved off for all samples probing interaction with importin-α.

A description of the conformational ensemble of full length B/NP was obtained using the crystal structure of the folded domain (PDB 3TJ0^23^) and Flexible-Meccano^30^ to add the intrinsically disordered tail as a conformational ensemble. The backbone dihedral angle distribution identified by a Flexible-Meccano/ASTEROIDS^31^ combination to describe B/NP_TAIL_ (see above) was assumed to be valid also within the full length protein construct. 200 conformers were generated and SAXS curves for each of the conformers were calculated using CRYSOL^69^ and averaged to obtain the expected SAXS curve for the full length B/NP ensemble.

### Isothermal titration calorimetry studies

B/NP_TAIL_ and importin-α7 were dialyzed in the same buffer (20 mM Tris-HCl pH 7.5; 150 mM NaCl) before the titration. The ITC titration experiments were done at 20 °C using a MicroCal ITC200 (GE Healthcare) with 16 × 2.4 µL injections of 150 µM importin-α7 into a 20 µM B/NP_TAIL_ solution. Integration of the titration curves was performed using the ORIGIN software (OriginLab, Northampton, United Kingdom) to extract thermodynamic parameters, stoichiometry N, equilibrium association constant K_a_ and the binding enthalpy ΔH. The Gibbs free energy of binding ΔG was calculated from the relation ΔG = −RT ln(K_a_) and the binding entropy ΔS was deduced from the equation (ΔG = ΔH − TΔS). All titrations fit the single-binding site mechanism with 1:1 stoichiometry and binding parameters were calculated as the average of three independent experiments ± SD.

## Electronic supplementary material

Supplementary Information
