# Supplementary material for: Structural analysis of the complex between influenza B nucleoprotein and human importin-α
Source: Sci Rep. 2017 Dec 7;7:17164. doi: 10.1038/s41598-017-17458-z (PMC5719345; doi:10.1038/s41598-017-17458-z)
Supplement: Supplementary file 1 — Supplementary Information [file 41598_2017_17458_MOESM1_ESM.pdf]

Structural analysis of the complex between influenza B nucleoprotein and human importin-α

Alice LABARONNE[1$], Sigrid MILLES[1$], Amélie DONCHET[1], Malene Ringkjøbing JENSEN[1], Martin BLACKLEDGE[1], Jean-Marie BOURHIS[1], Rob WH RUIGROK[1] & Thibaut CREPIN[1*]

[1] Univ. Grenoble Alpes, CNRS, CEA, IBS, F-38000 Grenoble

Supplementary information

Supplementary Figure 1

Conformational propensities of B/NP$_{TAIL}$. (**a**) Local conformational propensities of a set of 200 conformers describing the structural propensities of B/NP$_{TAIL}$ as derived from a combination of Flexible-Meccano and ASTEROIDS using chemical shifts (grey bars) of B/NP$_{TAIL}$ and propensities as expected from a statistical coil (black lines). Categories as described in Fig. 2: β-sheet (βS), poly-proline (βP), right (αR) and left handed (αL). (**b**) Secondary chemical shifts as obtained from B/NP$_{TAIL}$ assignment (grey bars) and back calculated from the ASTEROIDS ensemble (red lines).

Supplementary Figure 2

Interaction of B/NP with importin-α7. SDS-PAGE gels (Tris-Glycine, 4-20 % polyacrylamide) stained with coomassie blue of the control experiments done on (**a**) a Superdex™ 200 increase 10/300GL column for B/NP alone, importin-α7 alone and B/NP$_{CORE}$ alone and on (**b**) a Superdex™ 75 10/300GL column for B/NP$_{TAIL}$ and importin-α7.

Supplementary Figure 3

Homogeneous sample analysis of recombinant B/NP$_{CORE}$.

The MALLS run was performed using a Superdex™ 200 increase 10/300GL (GE Healthcare). Sample injection and buffer flow was controlled by a Hitachi L2130 pump, following the SEC column was a L-2400 UV detector (Hitachi), Optilab T-rEX refractometer (Wyatt technologies) and a DAWN HELEOS-II multi angle light scattering detector (Wyatt technologies). Prior to injection, columns and systems were equilibrated in 5 to 10 column volumes of running buffer 20 mM Tris-HCl pH 7.5, 150 mM NaCl and 5 mM β-ME. A 50 µL injection was performed using a recombinant B/NP$_{CORE}$ sample concentrated at 3.2 mg.mL^{-1} with a constant flow rate of 0.5 mL.min^{-1}. Accurate MALLS mass prediction was performed with the Astra software (Wyatt Technologies). Curves were represented with Graphpad

(Prism). The SEC-MALLS chromatograms with the UV signal is shown as a backtrace and molecular weight (red) estimate below the peak.

Supplementary Figure 4

Rg determination by Guinier extrapolation. Guinier plots of the scattering curves are shown with a line of best fit and residuals for, (**a**) importin-α7, (**b**) B/NP, (**c**) the B/NP_{TAIL}:importin-α7 complex and (**d**) the B/NP:importin-α7 complex. The slopes of fits are equal to $-Rg^2/3$, and the vertical intercept is equal to the log of the zero-angle scattering intensity I(0). The maximum q in the Guinier fits was determined for qRg values less than 1.3. All fits residuals (displayed in the bottom panel) are randomly distributed around zero.

Supplementary Figure 1

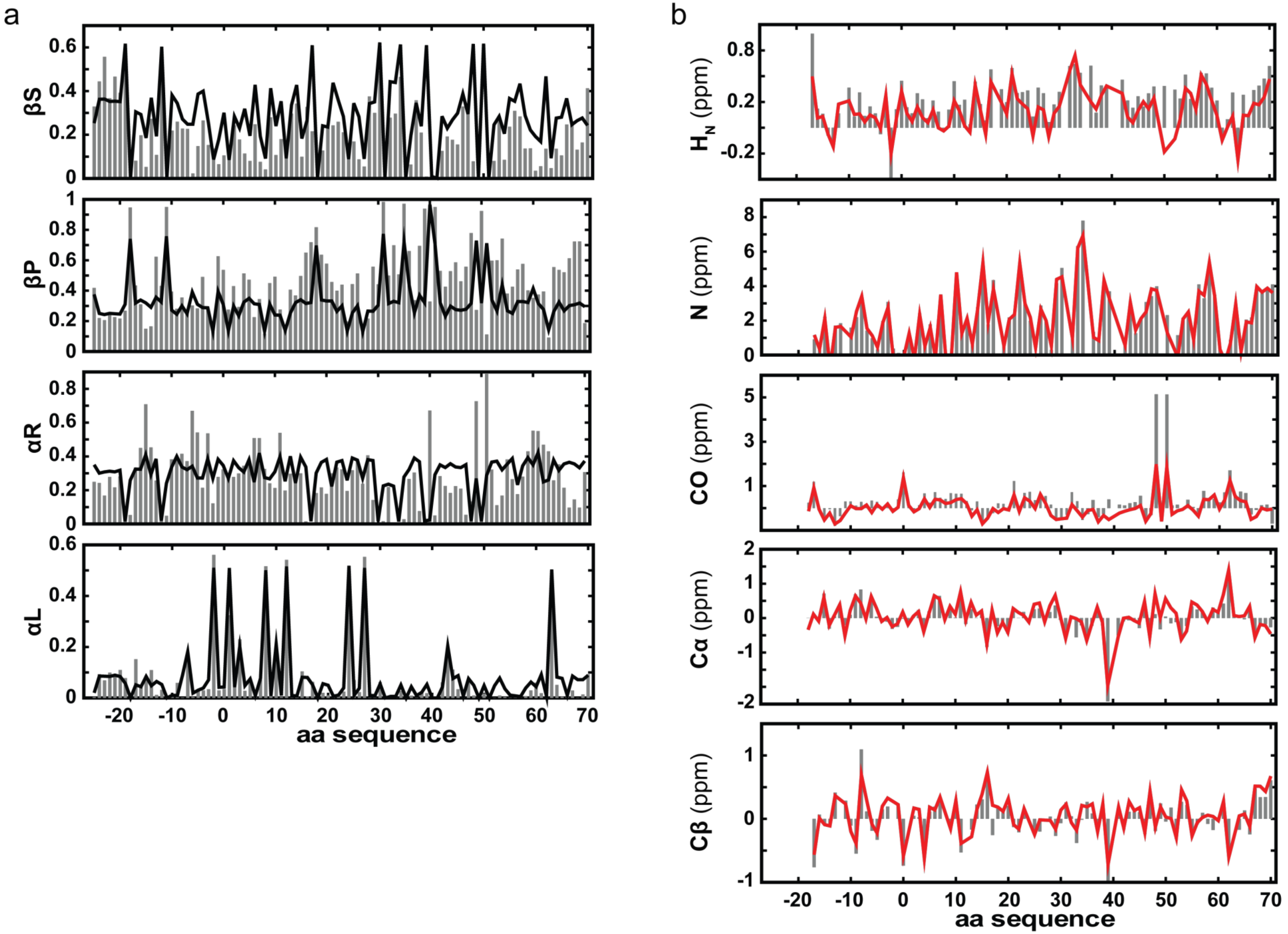

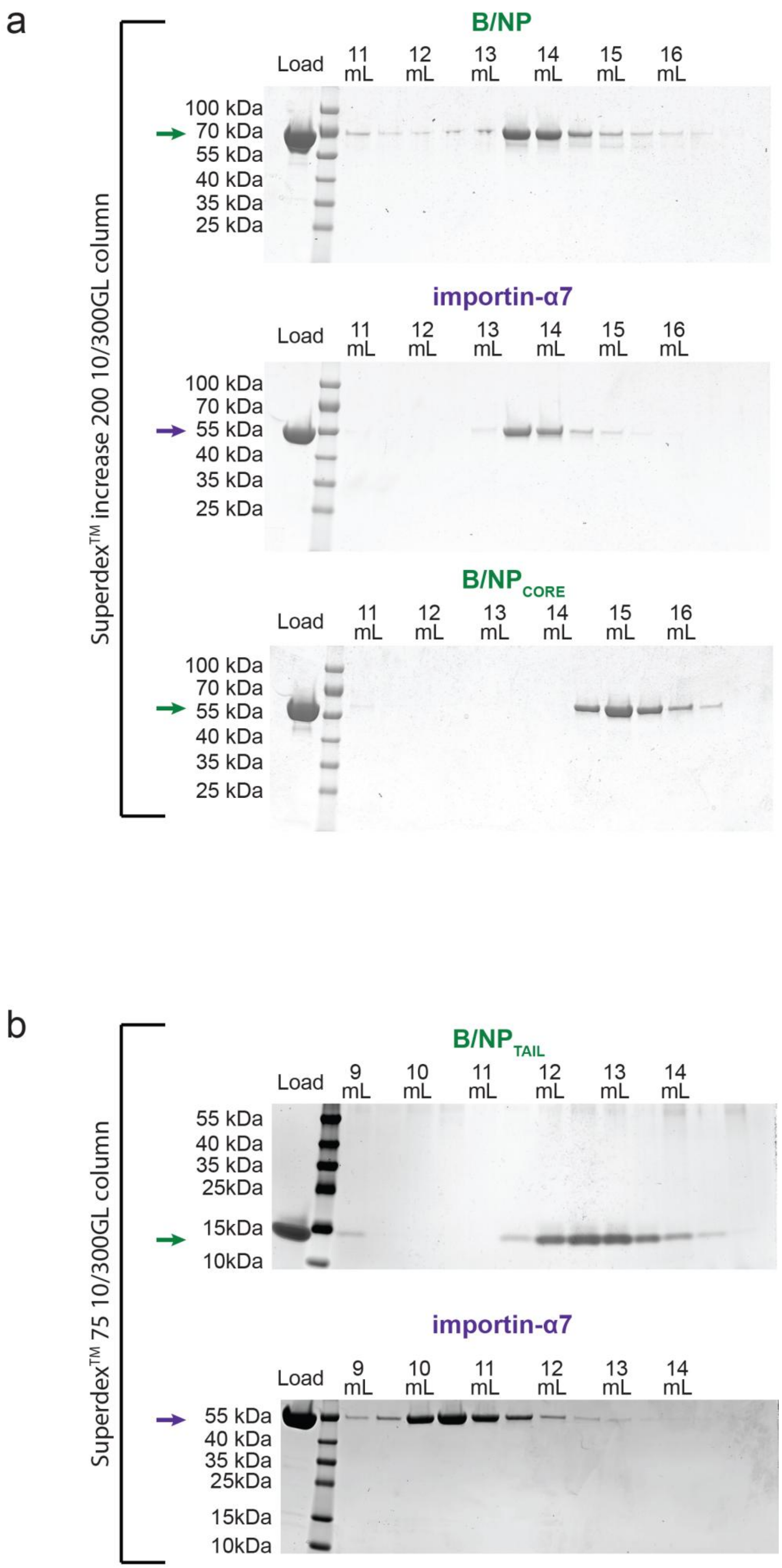
a
Superdex™ increase 200 10/300GL column
B/NP
Load
11 mL
12 mL
13 mL
14 mL
15 mL
16 mL
100 kDa
70 kDa
55 kDa
40 kDa
35 kDa
25 kDa
importin-α7
B/NPCORE
b
Superdex™ 75 10/300GL column
B/NPTAIL
9 mL
10 mL
15kDa
10kDa
25kDa

Supplementary Figure 3

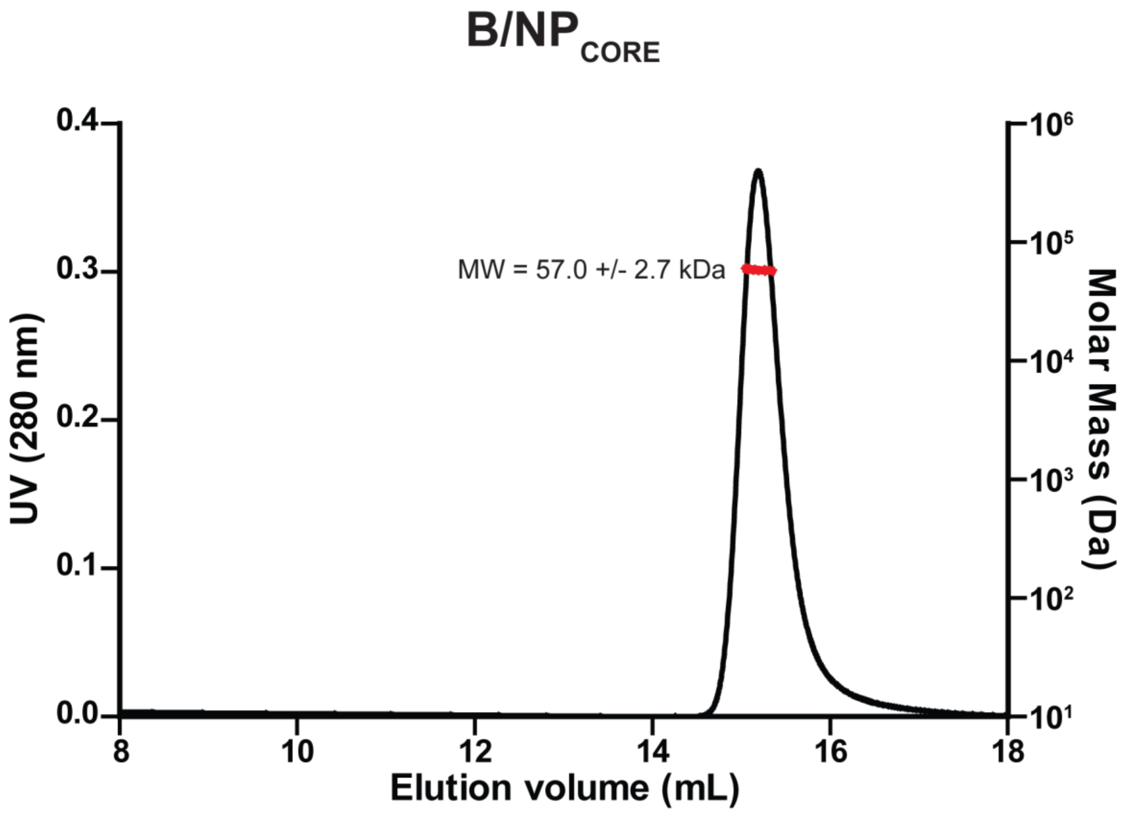

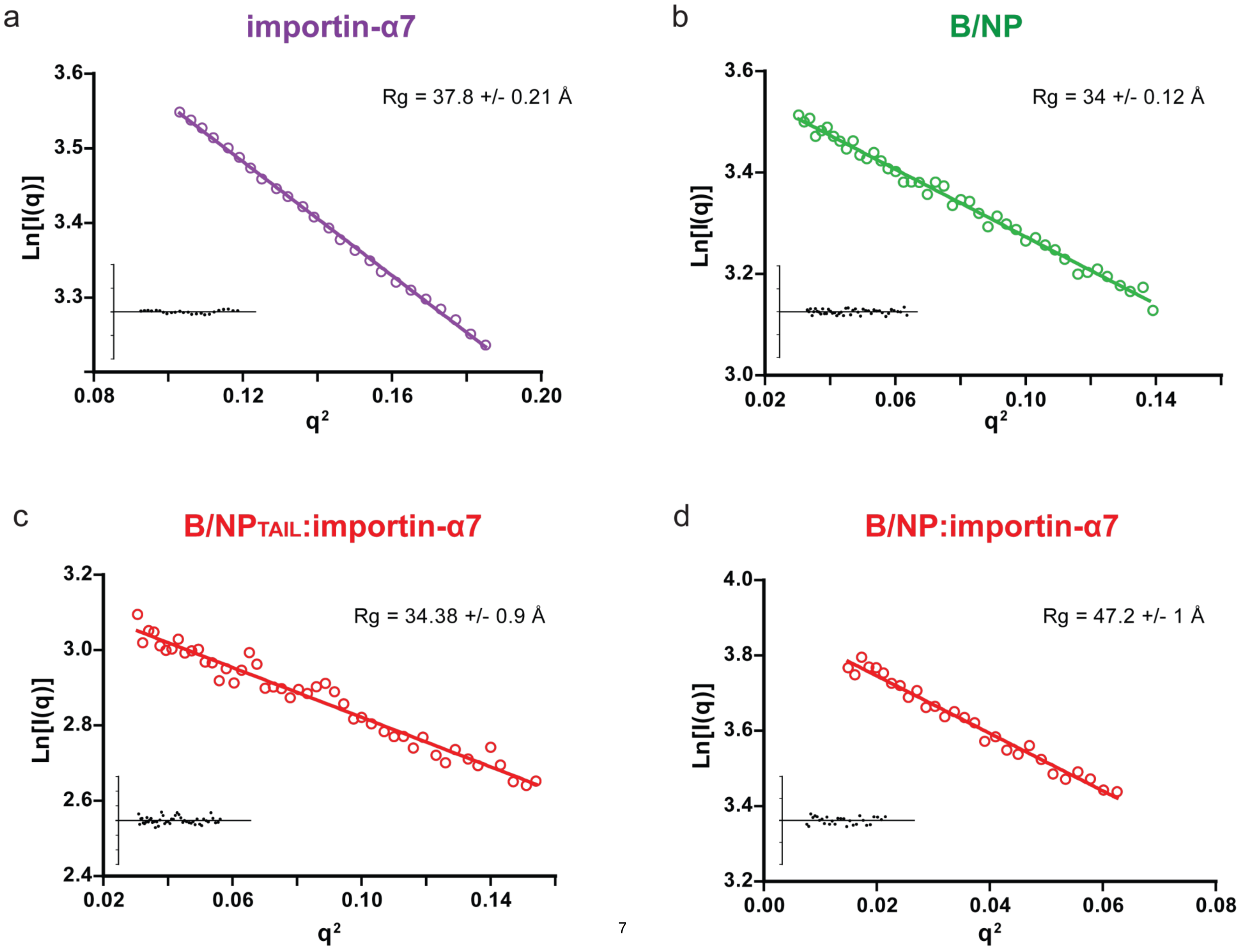

a
importin-α7
Rg = 37.8 +/- 0.21 Å
Ln[I(q)]
3.6
3.5
3.4
3.3
0.08
0.12
0.16
0.20
q²
b
B/NP
Rg = 34 +/- 0.12 Å
Ln[I(q)]
3.6
3.4
3.2
3.0
0.02
0.06
0.10
0.14
q²
c
B/NPTAIL:importin-α7
Rg = 34.38 +/- 0.9 Å
Ln[I(q)]
3.2
3.0
2.8
2.6
2.4
0.02
0.06
0.10
0.14
q²
d
B/NP:importin-α7
Rg = 47.2 +/- 1 Å
Ln[I(q)]
4.0
3.8
3.6
3.4
3.2
0.00
0.02
0.04
0.06
0.08
q²
